# Effects of Approach-Avoidance Training on the Extinction and Return of Fear Responses

**DOI:** 10.1371/journal.pone.0131581

**Published:** 2015-07-22

**Authors:** Angelos-Miltiadis Krypotos, Inna Arnaudova, Marieke Effting, Merel Kindt, Tom Beckers

**Affiliations:** 1 Department of Clinical Psychology, University of Amsterdam, Amsterdam, the Netherlands; 2 Amsterdam Brain and Cognition Center, Amsterdam, the Netherlands; 3 Department of Psychology, KU Leuven, Leuven, Belgium; Swansea University, UNITED KINGDOM

## Abstract

**Background and Objectives:**

Exposure therapy for anxiety involves confronting a patient with fear-evoking stimuli, a procedure based partially on Pavlovian extinction. Exposure and other extinction-based therapies usually lead to (partial) reduction of fear symptoms, but a substantial number of patients experience a return of fear after treatment. Here we tested whether the combination of fear extinction with modification of approach-avoidance tendencies using an Approach-Avoidance Task (AAT) would result in the further reduction of conditioned fear and/or help prevent return of fear after extinction.

**Methods:**

Two groups of participants underwent a fear acquisition procedure during which pictures of one neutral object were sometimes paired with shock (CS^+^), whereas pictures of another neutral object were not (CS^−^). The next day, in a fear extinction procedure, both objects were presented without shock. During the subsequent joystick AAT, one group primarily pulled CS^+^ pictures towards themselves and pushed CS^−^ pictures away from themselves; reversed contingencies applied for the other group.

**Results:**

Approach training was effective in modifying conditioned action tendencies, with some evidence for transfer to a different approach/avoidance task. No group differences in subjective fear or physiological arousal were found during subsequent post- training and return-of-fear testing.

**Limitations:**

No reliable return-of-fear was observed in either group for either subjective or physiological fear measures.

**Conclusions:**

Our results suggest that approach training may be of limited value for enhancing the short- and long-term effects of extinction-based interventions.

## Introduction

Exposure therapy is one of the most effective interventions for anxiety disorders [1]. Nonetheless, the effects of exposure therapy are often incomplete (no full elimination of anxiety symptoms) or short-lived (symptoms resurface after seemingly successful therapy; [2]). Identifying and remedying the limitations of exposure therapy is critical if we want to increase its short-term (therapeutic efficacy) and long-term (prevention of relapse) effectiveness.

Insights in that direction can be gleaned from experimental psychopathology research. The laboratory parallel of exposure therapy is Pavlovian fear extinction (we stress that although a commonly used paradigm that mimics exposure therapy, extinction is not the only mechanism that can account for exposure therapy outcomes [3]). After a neutral cue (e.g., a picture of a geometrical figure; Conditioned Stimulus or CS) has been paired with an aversive event (e.g., a shock; Unconditioned Stimulus or US), such that presentation of the CS comes to elicit conditioned fear responses (e.g., subjective apprehension, physiological arousal), repeated presentations of that CS without the US will typically result in the reduction of those conditioned fear responses (fear extinction). However, following fear extinction, fear responses can be made to return through simple experimental manipulations, such as the unsignaled presentation of the US (i.e., reinstatement; [4]). Reinstatement is regarded as a laboratory model for return of fear and relapse after treatment. Of importance, following Pavlovian extinction, acquired negative CS evaluations reduce only slightly or even remain unaffected despite the reduction in US-expectancy levels [5]. It has been suggested that lingering negative CS evaluations after fear extinction might play a crucial role in promoting the eventual return of conditioned fear [6]. Therefore, the modification of CS evaluations might help to enhance fear reduction after extinction and possibly prevent the return of fear.

A way to assess and manipulate stimulus evaluations is via Approach-Avoidance Tasks (AAT; [7]). These are reaction time tasks that tap into the bidirectional link between stimulus evaluation (i.e., pleasant versus unpleasant) and behaviour (i.e., approach versus avoidance), as people have a behavioural tendency to pursue pleasant stimuli and stay away from unpleasant stimuli [8, 9]. Accordingly, in *assessment* AATs, in which participants are instructed to approach and avoid stimuli with different hedonic value, reaction times (RT) are typically shorter for approaching pleasant and avoiding negative cues than for the reverse. In *training* AATs, participants are made to primarily approach one type of stimulus and avoid another type of stimulus, as a result of which they become faster in approaching the former and avoid the latter than vice versa and also come to prefer the former over the latter stimuli in explicit [10] and implicit assessments [11]. From those results, it may be anticipated that approach-avoidance training would also help to alter negative evaluations towards conditioned fear cues. If that is the case, then it could be expected that the fear-reducing effect of extinction training, which arguably targets mainly the US-expectancy elicited by a fear cue, could be enhanced by supplementing it with a training AAT to target the evaluative connotation of that fear cue. Of note, in an alcoholic patient sample, the combination of cognitive-behaviour therapy with avoidance training of alcohol cues using an AAT resulted in lower relapse rates compared to controls ([12]; see [13], and [14], for more examples of training AATs), consistent with the idea that modifying stimulus evaluations through approach-avoidance training might help to enhance treatment effects and/or counter relapse.

Given those encouraging findings, we hypothesized that the addition of a training AAT to Pavlovian extinction might lead to enhanced fear reduction and/or diminished return of fear, with results having potential implications for extinction-based therapies.

In the present study, participants underwent a fear acquisition procedure during which pictures of one neutral object (CS^+^) were always followed by an electric shock, whereas pictures of another neutral object (CS^−^) were never followed by shock. An extinction procedure followed the next day, during which both CSs were repeatedly presented without shock. Next, participants performed a joystick version of the AAT (*training* AAT). During this task, participants had to push or pull a joystick in response to the tilt of the presented white frame that surrounded a picture of either the CS^+^ or the CS^−^. Contingencies between the tilt of the frame and the identity of the picture were such that one group of participants had to primarily pull the joystick (approach) in response to CS^+^ pictures and push the joystick (avoid) in response to CS^−^ pictures (Approach CS^+^ group); the other participants were made to do the opposite (Avoid CS^+^ group). Afterwards, in the post-training test phase, both CSs were again presented without any of them being followed by shock. Lastly, we tested the reinstatement of fear responses.

Conditioned fear responses were assessed through subjective ratings (trial-by-trial US-expectancy ratings), fear-potentiated startle responses (FPS) and skin conductance responses (SCR). To assess transfer of approach-avoidance training beyond the joystick AAT, we measured approach-avoidance tendencies towards the CSs at the end of the experiment using a manikin version of the AAT (*assessment* AAT) in which participants had to move a manikin on screen towards or away from CS^+^ and CS^−^ pictures. We also measured subjective CS^−^ evaluations on rating scales.

We expected that the approach training contained in the joystick AAT would attenuate negative evaluations of the CS^+^ relative to the CS^−^ in the approach CS^+^ group, leading to reduced differential evaluations in that group compared to the avoid CS^+^ group. We expected such reduction, or possible reversal, in negative evaluation of the CS^+^ relative to the CS^−^ to also influence subjective ratings of the CSs and responding in the manikin AAT, such that relative to the avoid CS^+^ group, participants in the approach CS^+^ group would be faster to make the manikin approach the CS^+^ and avoid the CS^−^. If changing CS evaluations through approach training affects the reduction and/or return of fear, we should observe differences in differential fear responding (FPS, SCR, US-expectancy) between both training groups after approach training (i.e., during the post-training and/or the recovery testing phase), reflecting enhanced fear reduction and/or attenuated return-of-fear in the approach CS^+^ group compared to the avoid CS^+^ group.

## Materials and Methods

### Participants

Forty-five individuals participated for course credits or €7. Five individuals with incomplete data (e.g., not attending the second day) were excluded from the analyses. This resulted in 40 individuals (21 females; Mean age: 22.25 years), randomly assigned to the Approach CS^+^ (n = 22) or the Avoid CS^+^ (n = 18) group. The ethics committee of the University of Amsterdam has approved the study (EC number: 2013-COP-2952) and written informed consent was provided from all participants.

### Apparatus and materials

#### 0.0.1 Stimuli

During all conditioning phases (i.e., acquisition, extinction, post-training and reinstatement test), pictures (50 mm × 50 mm) of a cube and a cylinder, each depicted from four different viewpoints, served as CS^+^ or CS^−^. The assignment of the shapes (cube versus cylinder) to the role of CS^+^ or CS^−^ was random. All pictures were presented against a white (100 mm × 100 mm) frame.

Electrical stimulation (i.e., shock) of 2-ms duration, delivered through a pair of Ag electrodes, served as the US. Standard conductive gel was applied between the electrodes and the skin.

For both the joystick and the manikin AATs, the same pictures were used as during the conditioning phases. For the joystick AAT, however, the surrounding white frame (100 mm × 100 mm) was tilted 3° to the left or to the right. For the manikin AAT, all pictures were presented against a landscape (100 mm × 55 mm) or portrait (55 mm × 100 mm) white frame. A white manikin figure (71 mm × 95 mm) was also presented during the manikin AAT (see below).

#### Self-reports and US-evaluations

We measured anxiety sensitivity with the Anxiety Sensitivity Index (ASI; [15]) and state and trait anxiety with the State and Trait Anxiety Inventories (STAI-S/STAI-T; [16]). Participants also evaluated US pleasantness (from -5, *unpleasant*, to +5, *pleasant*), US intensity (*weak*, *moderate*, *intense*, *enormous*, *unbearable*), and US startlingness (*not*, *light*, *moderate*, *strong*, *too strong*).

#### US-expectancy ratings

Online US-expectancy ratings were recorded on a scale from -5 (*certainly no electric stimulus*) to +5 (*certainly an electric stimulus*). Participants rated their expectancy of electric stimulus occurrence, within 7 s after CS onset, by moving the cursor on a computer scale and by clicking the left button of a standard computer mouse.

#### Fear potentiated startle

FPSs were assessed by electromyography (EMG) on the left orbicularis oculi muscle. We evoked the startle response by delivering a loud noise (40 ms; 104 dB) through closed headphones. A 70-dB broadband background noise was continuously administered.

For measuring the fear potentiated startle (FPS), two Ag/AgCl electrodes (5-mm) filled with standard electrolyte gel were positioned approximately 1 cm under the pupil and 1 cm below the lateral canthus. The ground electrode was placed 1 cm below the hairline on the forehead approximately [17]. The EMG signal was amplified in two stages. The characteristics of the input stage were: 1) an input resistance of 10 M Ω, 2) a frequency response of DC-1500 Hz, and 3) an amplification factor of 200. Unwanted interference was reduced by using a 50-Hz notch filter. The second stage amplified the signal with a variable amplification factor of 0–100 x. The raw EMG signal was sampled at 1000 Hz and band-pass filtered (28–500 Hz, Butterworth, 4th order).

#### Skin conductance response

Skin conductance responses (SCR) were measured using two electrodes attached to the medial phalanges of the first and second fingers of the non-preferred hand.

For the measurement of the skin conductance responses (SCR), we used an input device with a sine shaped excitation voltage (±.5 V) of 50 Hz, derived from the mains frequency. The input device was connected to two Ag/AgCl electrodes (20 mm × 16 mm. The signal from the input device was led through a signal-conditioning amplifier and the analogue output was digitized at 100 Hz by a 16-bit AD-converter (National Instruments, NI-6224).

#### CS evaluations

Participants rated the valence of each CS picture on a scale from -5 (negative) to + 5 (positive).

### Experimental procedure

**Fig 1:**
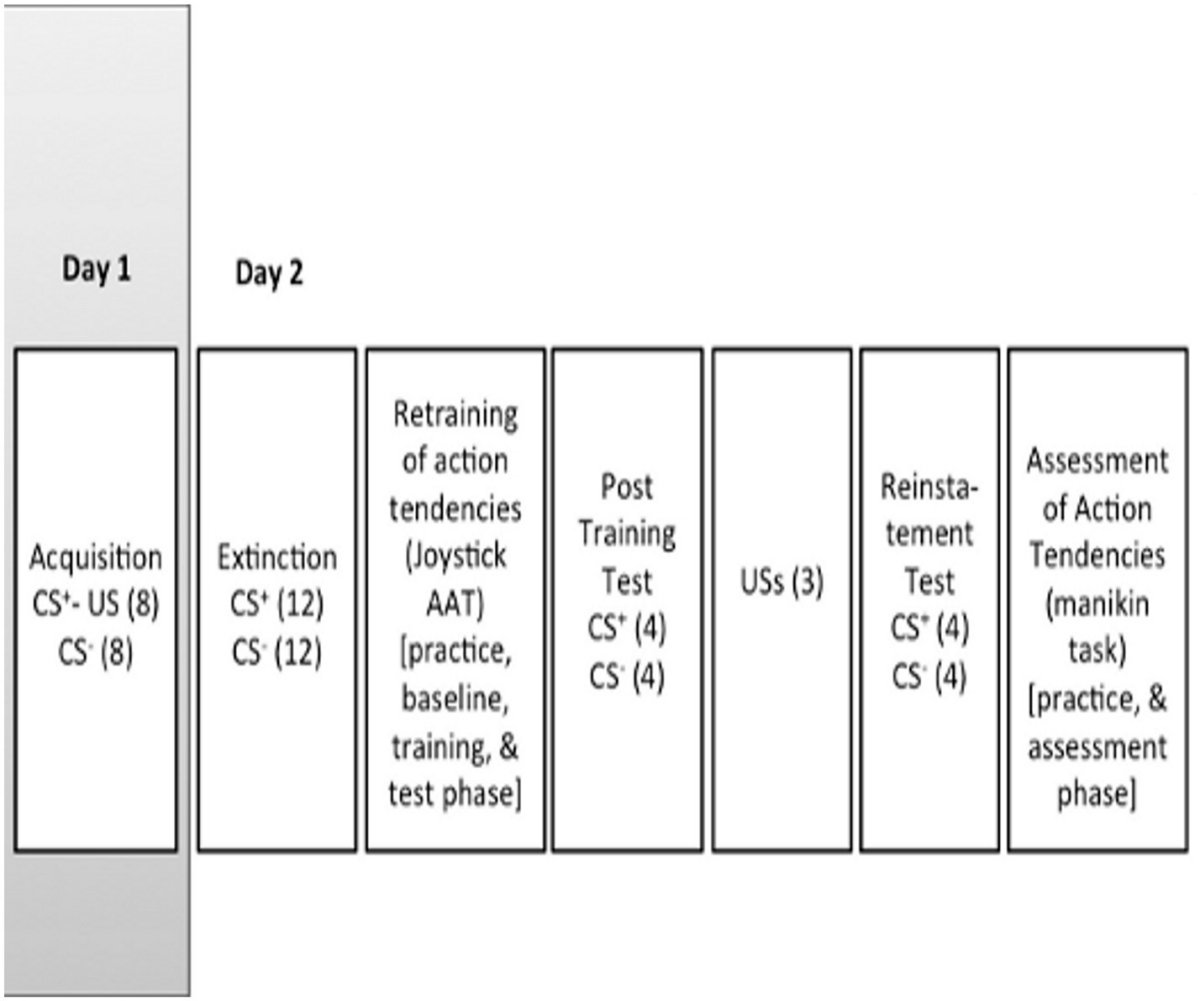
Graphical depiction of the experimental phases for day 1 (grey area) and day 2 (white area) (see text for details).

The experiment took place on two consecutive days see Fig 1. On day one, participants underwent a fear *acquisition* procedure. On day two, participants underwent an *extinction* procedure, a *training AAT* (joystick task), a *post-training test phase* and a *reinstatement test* phase as well as an *assessment AAT* (manikin task).

The acquisition, extinction and post-training test phase started with 10, 10 and 6 startle habituation trials, respectively. We presented isolated startle probes (Noise Alone; NA trials) throughout all conditioning phases as well.

On-screen and oral instructions indicated that pictures of two objects would be presented with pictures of one object sometimes followed by an electric stimulus and pictures of the other object never followed by an electric stimulus. Participants were instructed to rate their US expectancies on each trial, during all conditioning phases.

#### Preparation and acquisition

On day one, participants read the information brochure and signed the informed consent. Afterwards, they filled in the STAI-S. Next, the EMG, SCR and shock electrodes were attached and the US was set to a level indicated by participants to be ‘uncomfortable but not painful’.

Each CS was presented 8 times. Each trial lasted 8 s. A startle probe was presented 7 s after CS onset on each trial. During the acquisition trials, a US followed 7.5 s after onset of every CS^+^. Inter-trial intervals (ITIs) were 15, 20, or 25 s, with a mean of 20s. In case of a NA trial (8 trials in total), the startle probe was presented alone, followed by an 8-sec interval and the subsequent ITI.

At the end of acquisition, participants were asked to remember the CS^−^ US contingencies for the following day.

#### Extinction

Extinction consisted of 12 unreinforced presentations of each CS (3 times each per viewpoint) and 12 NA trials, with similar parameters as for acquisition.

#### Training AAT

Modelled after previous approach-avoidance training studies (e.g., [18]), the joystick AAT consisted of four phases: *practice*, *baseline*, *training* and *test*. During the practice phase, participants were instructed to push or pull a joystick in response to the tilt of the white frame presented on screen (left or right), with the frame shrinking or enlarging in response to the joystick response, respectively. Assignment of picture format (tilted to left or right) to response (push or pull) was counterbalanced across participants. In case of an incorrect response, participants repeated the same trial until a correct response was given [18]. Next, the baseline phase (32 trials) began during which participants had to push and pull the joystick equally often for both CSs (i.e., CS^+^ and CS^−^ pictures were presented tilted to the left equally often as to the right). From that phase onwards, the pictures of the CSs and the surrounding frame would expand or shrink in accordance to participants’ movements (i.e., shrink upon a push response and enlarge upon a pull response).

Unbeknownst to participants, the training phase followed immediately afterwards. In the Approach CS^+^ group, contingencies between picture content (CS^+^ or CS^−^) and picture format (tilted left or right) were arranged such that participants had to pull the joystick in response to the CS^+^ 89% of the times and push the joystick 11% of the times, with the opposite contingencies for the CS^−^. The reversed contingencies applied for the Avoid CS^+^ group. The training phase consisted of 448 trials, equally divided into 2 blocks of trials, with a short break in between.

The task ended with a test phase that was identical to the baseline phase.

#### Post-training test

After a 4-min break, we tested fear responses in the post-training test phase by presenting four unreinforced trials for each CS (intermixed with four NA trials).

#### Reinstatement test

For evoking reinstatement, 3 unsignaled shocks were administered. The reinstatement test phase consisted of four unreinforced presentation of each CS along with four NA trials.

Electrodes for shock administration, FPS measurement and SCR measurement were removed before the start of the manikin AAT.

#### Action tendencies assessment

The manikin AAT consisted of two blocks of 20 trials each (4 practice trials and 16 test trials; [19]). For the practice trials, 2 vertical and 2 horizontal grey frames were presented. For the test trials, each CS viewpoint was presented twice in semi-random order (no more than two CS^+^ or CS^−^ trials in a row). On each trial, a white manikin figure appeared centred to the bottom or top half of a black computer screen. After 1500 ms, a grey frame (practice trials) or white frame with CS picture (test trials) was presented, centred to the opposite half of the screen. Participants were instructed to move the manikin according to the orientation of the frame (e.g., toward landscape and away from portrait or vice versa), with instructions switched between blocks. Participants could move the manikin upward or downward by pressing the “Y” (labelled “↑”) or “B” (labelled “↓”) key, respectively, on a standard computer keyboard. The order of instructions was counterbalanced between participants. Upon a response, the manikin started moving, to disappear after 2000 ms. In case of an incorrect response, a red cross appeared at the manikin’s starting position for 500 ms. The ITIs were fixed to 2000 ms.

#### CS and US evaluations and questionnaires

Lastly, participants rated the pleasantness of the CSs, filled in the STAI-T and ASI questionnaires, and rated the US.

### Data Reduction and Response Definition

Skin conductance responses were defined as the difference between the maximum skin conductance value during the first seven seconds after CS onset and the average baseline period (i.e., 1 s before CS onset). The raw absolute SCR scores were square root transformed; in case of a negative raw value the negative sign was then re-applied [20].

EMG responses were defined as the peak amplitude by analysing the first derivative of the processed signal in the interval of 0–250 ms following probe presentation.

For both the joystick and the manikin AAT, incorrect trials, test trials with reaction times exceeding 3000 ms (combined scores, 5.59% and 5.23% of the joystick and the manikin trials, respectively), practice trials, as well as the training block of the joystick AAT, were omitted from the analyses. We then calculated median reaction times (RTs) for each CS (CS^+^, CS^−^) by response type (approach/pull, avoid/push) combination for the manikin AAT as well as for each of the baseline and test phases of the joystick AAT.

### Statistical analyses

Ratings for the US and CS evaluations were analysed using separate Analyses of Variance (ANOVAs). US-expectancy ratings, SCR and FPS responses were analysed for each phase with separate mixed-measures ANOVAs with Type of Stimulus (CS^+^, CS^−^) as within-subjects factor and Group (approach CS^+^, avoid CS^+^) as between-subjects factor. A Greenhouse-Geisser procedure was used in case the assumption of sphericity was violated. In order to test for post-training effects and reinstatement, we performed separate mixed-measures ANOVAs, with Stimulus (CS^+^, CS^−^) and Phase (extinction, post-training or extinction, reinstatement test) as within-subjects factors and Group (approach CS^+^, avoid CS^+^) as between-subjects factor.

For the joystick AAT, RTs were analysed with a mixed-measures ANOVA with Stimulus (CS^+^, CS^−^), Response (approach, avoid), and Phase (baseline, test) as within-subjects factors and Group (approach CS^+^, avoid CS^+^) as between-subjects factor. For the manikin AAT, RTs were analysed with a mixed-measures ANOVA with Stimulus (CS^+^, CS^−^) and Response (approach, avoid) as within-subjects factor and Group (approach CS^+^, avoid CS^+^) as between-subjects factor. We report and interpret only the results of the main interaction effects for both AATs, as the unequivocal interpretation of main effects in such tasks is not possible (see [19], supplementary material).

## Results

### Self-report data

Groups did not differ in terms of sex, state or trait anxiety, anxiety sensitivity, selected US level or US evaluations (see Table 1).

**Table 1:** Mean values (standard deviations in parenthesis) per group for demographic characteristics, STAI-S, STAI-T, ASI, selected US level, US characteristics and CS evaluations.

	Group			
Measures	Avoid CS^+^	Approach CS^+^	*t*-values	Pearson’s *χ* ^2^
Age	22,28 (2,14)	22,23 (3,34)	*t* < 1	
Sex	8 Females	13 Females		*χ* ^2^ = 0,85
STAI-S	32,78 (9,60)	33,82 (8,13)	*t* < 1	
STAI-T	33,28 (6,30)	36,86 (6,63)	*t*(38) = -1.74*	
ASI	12,89 (10,45)	13,05 (6,97)	*t* < 1	
Selected US level (in mA)	26,39 (14,81)	31,18 (15,01)	*t* < 1.05	
US pleasantness	-6,33 (2,30)	-6,18 (1,84)	*t* < 1	
US intensity	3,39 (0,85)	3,50 (0,67)	*t* < 1	
US startlingness	3,61 (0,92)	3,82 (0,80)	*t* < 1	
CS^+^ evaluation	-2,48 (1,66)	-1,61 (1,82)		
CS^−^ evaluation	2,90 (1,91)	2,04 (1,85)		

*Note*. STAI-S: State Anxiety Inventory; STAI-T: Trait Anxiety Inventory—Trait Measure; ASI: Anxiety Sensitivity Index; US: Unconditioned Stimulus (i.e., shock); CS: Conditioned Stimulus.

* *p* < .1.

### Training AAT data

**Fig 2:**
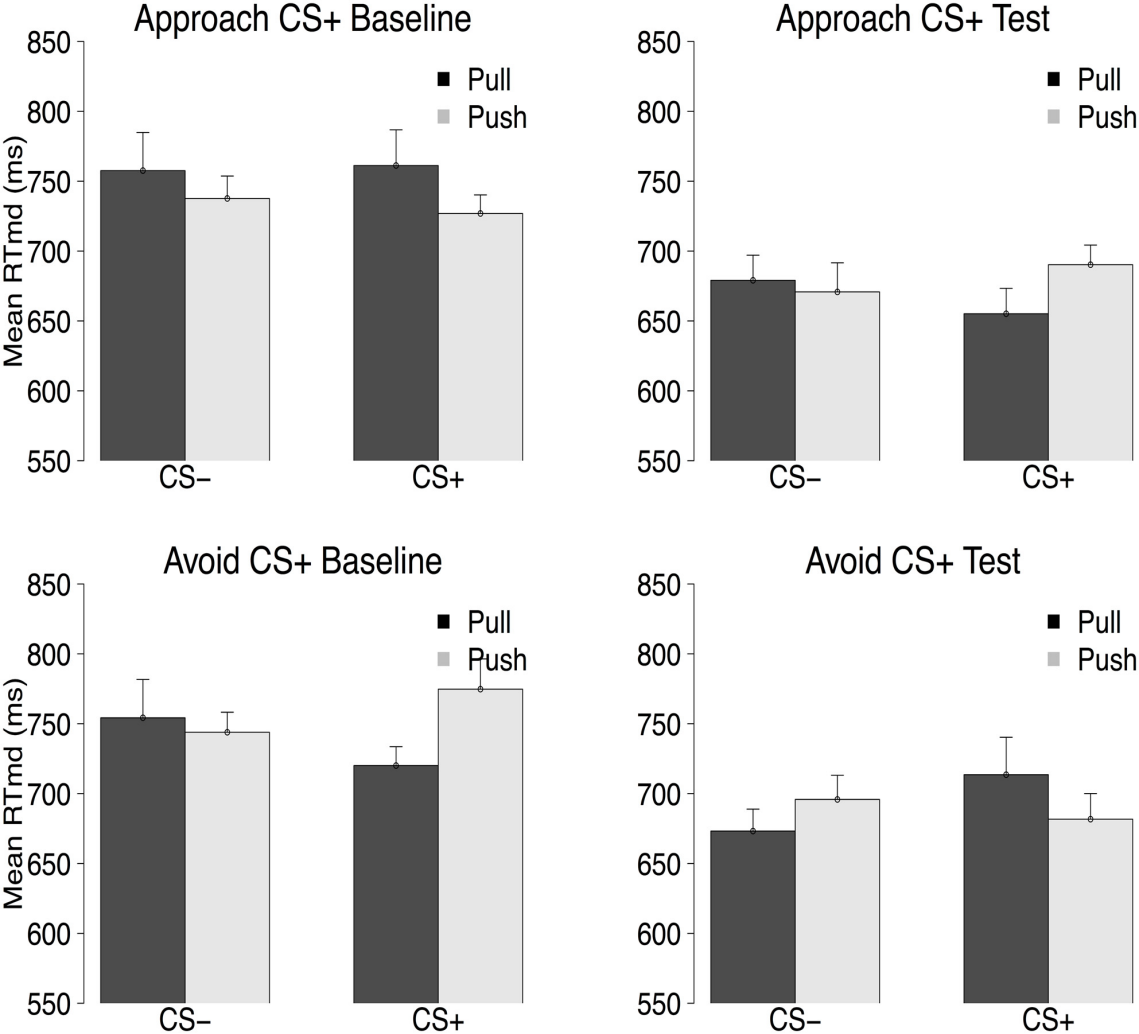
Mean median RT in ms for the Approach CS^+^ (top panels) and the Avoid CS^+^ (bottom panels) groups for the baseline (left panels) and the test block (right panels) of the joystick AAT. Error bars were computed using the approach described in [21].

In line with our hypothesis, results from the joystick AAT (see Fig 2) revealed that the between-groups difference in response pattern was different between the baseline and the test phase, Stimulus × Response × Phase × Group interaction, *F*(1, 38) = 8.97, *p* <.01, $\eta_{p}^{2}=.19$. We have previously demonstrated that fear conditioning results in the successful induction of action tendencies, as measured via an AAT (see Experiment 1 of [19]). However, as shown in Experiment 2 of [19], those tendencies are diminished following an extinction procedure. As such, no differences in action tendencies would be expected at the baseline block of the training AAT. Indeed, no Stimulus × Response interaction was observed for the assessment phase, *F*(1, 38) = 2.23, *p* = .14, $\eta_{p}^{2}=.06$, with the Stimulus × Response × Group interaction showing a non-significant trend, *F*(1, 38) = 3.57, *p* = .066, $\eta_{p}^{2}=.09$, that would, if anything, work against our hypothesis. No Stimulus × Response interaction was found for the test phase either, *F* < 1. Of importance, there was a significant Stimulus × Response × Group interaction, *F*(1, 38) = 5.13, *p* = .03, $\eta_{p}^{2}=.12$, which suggested, in line with our predictions, that participants in the approach CS^+^ group were faster to approach the CS^+^ and avoid the CS^−^ than the reversed and participants in the avoid CS^−^ group showing the opposite pattern. Taken together, our results indicate that the approach training was successful.

### US-expectancy rating data

**Fig 3:**
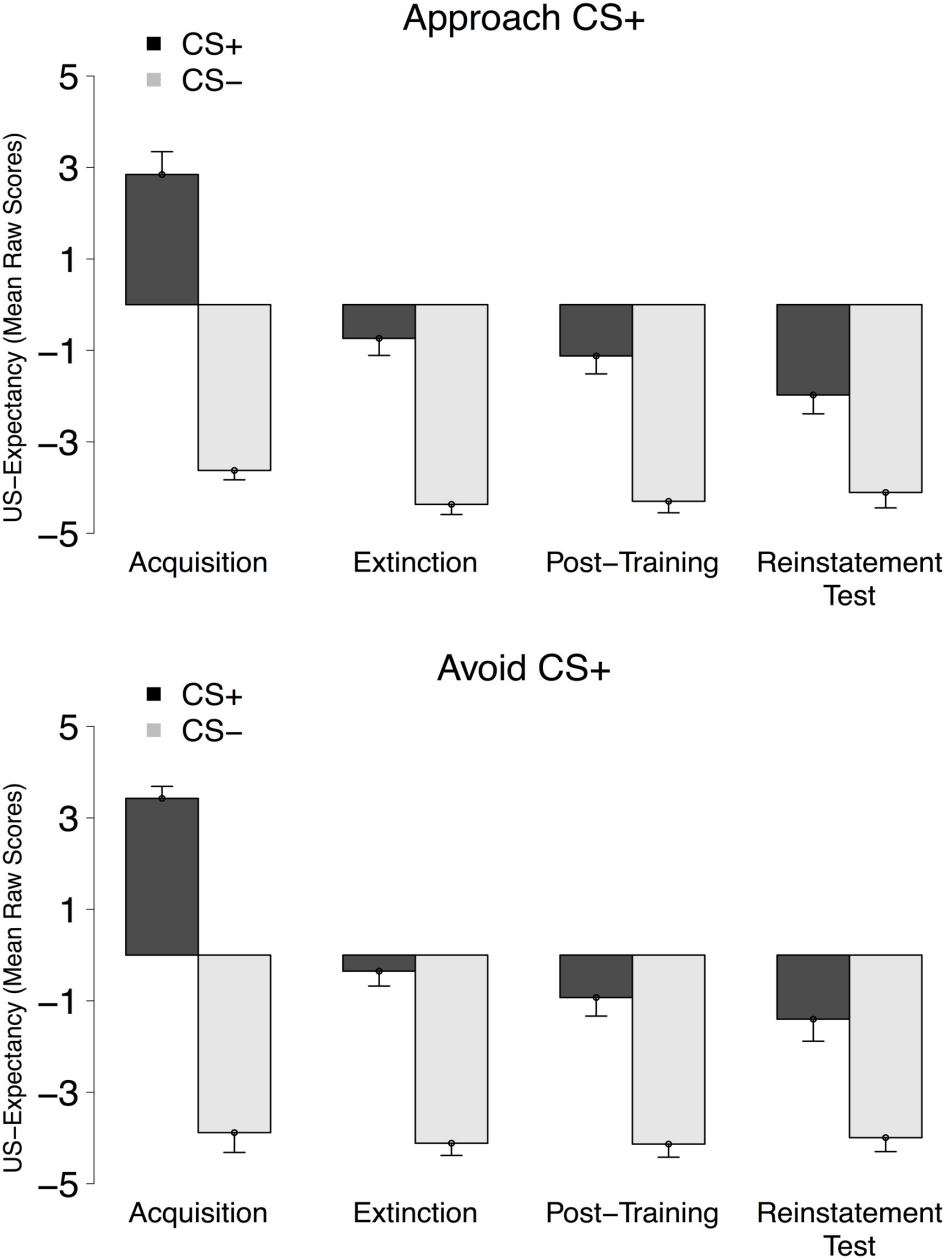
Mean US-expectancy scores of the Unconditioned Stimulus to the CS^+^ and CS^−^ during all the conditioning phases for the Approach CS^+^ (top panel) and the Avoid CS^+^ (bottom panel) group. Error bars were computed using the approach described in [21].

Mean US-expectancy ratings across the relevant conditioning phases are displayed in Fig 3. During the acquisition phase, participants in both groups learned to expect the US after the CS^+^ and not after the CS^−^, main effect of Stimulus, *F*(1, 38) = 360.79, *p* <.001, $\eta_{p}^{2}=.91$, Stimulus × Group interaction, *F* < 1.33, suggesting similarly successful acquisition of differential US-expectancy in both groups.

Differential US-expectancy was partially preserved in the extinction phase, main effect of Stimulus, *F*(1, 38) = 89.22, *p* <.001, $\eta_{p}^{2}=.70$, and this to a similar extent in both groups, Stimulus × Group interaction, *F* < 1. Nonetheless, compared to the acquisition phase, there was a significant decline in differential US-expectancy, Stimulus × Phase interaction, *F*(1, 38) = 37.39, *p* <.001, $\eta_{p}^{2}=.50$, across Groups, *F* < 1.

Differential US-expectancy seemed to further decline from the extinction to the post-training test phase, as suggested by a non-significant trend toward a Stimulus × Phase interaction, *F*(1, 38) = 3.44, *p* = .07, $\eta_{p}^{2}=.08$, which was comparable between groups, Stimulus × Phase × Group interaction, *F* < 1. Specifically, there was a non-significant trend toward a reduction in US-expectancy for the CS^+^, CS^+^ main effect of Phase, *F*(1, 38) = 3.60, *p* = .07, $\eta_{p}^{2}=.09$, and no change for the CS^−^, CS^−^ main effect of Phase, *F* < 1. Differential US-expectancy remained present throughout the post-training test phase, main effect of Stimulus, *F*(1, 38) = 53.44, *p* <.001, $\eta_{p}^{2}=.58$, for both groups, Stimulus × Group, *F* < 1.

Differential US-expectancy again declined from the extinction to the reinstatement test phase, Stimulus × Phase interaction, *F*(1, 38) = 23.39, *p* <.001, $\eta_{p}^{2}=.38$, in a similar fashion for both groups, Stimulus × Phase × Group interaction, *F* < 1. This change again resulted from a reduction in US-expectancy for the CS^+^, Stimulus × Phase interaction, *F*(1, 38) = 20.44, *p* <.001, $\eta_{p}^{2}=.35$, with no change for the CS^−^, Stimulus × Phase interaction, *F* < 1. Lastly, differential US-expectancy remained present in the reinstatement test phase, main effect of Stimulus, *F*(1, 38) = 23.82, *p* <.001, $\eta_{p}^{2}=.39$, similarly for both groups, Stimulus × Group interaction, *F* < 1.

### FPS data

**Fig 4:**
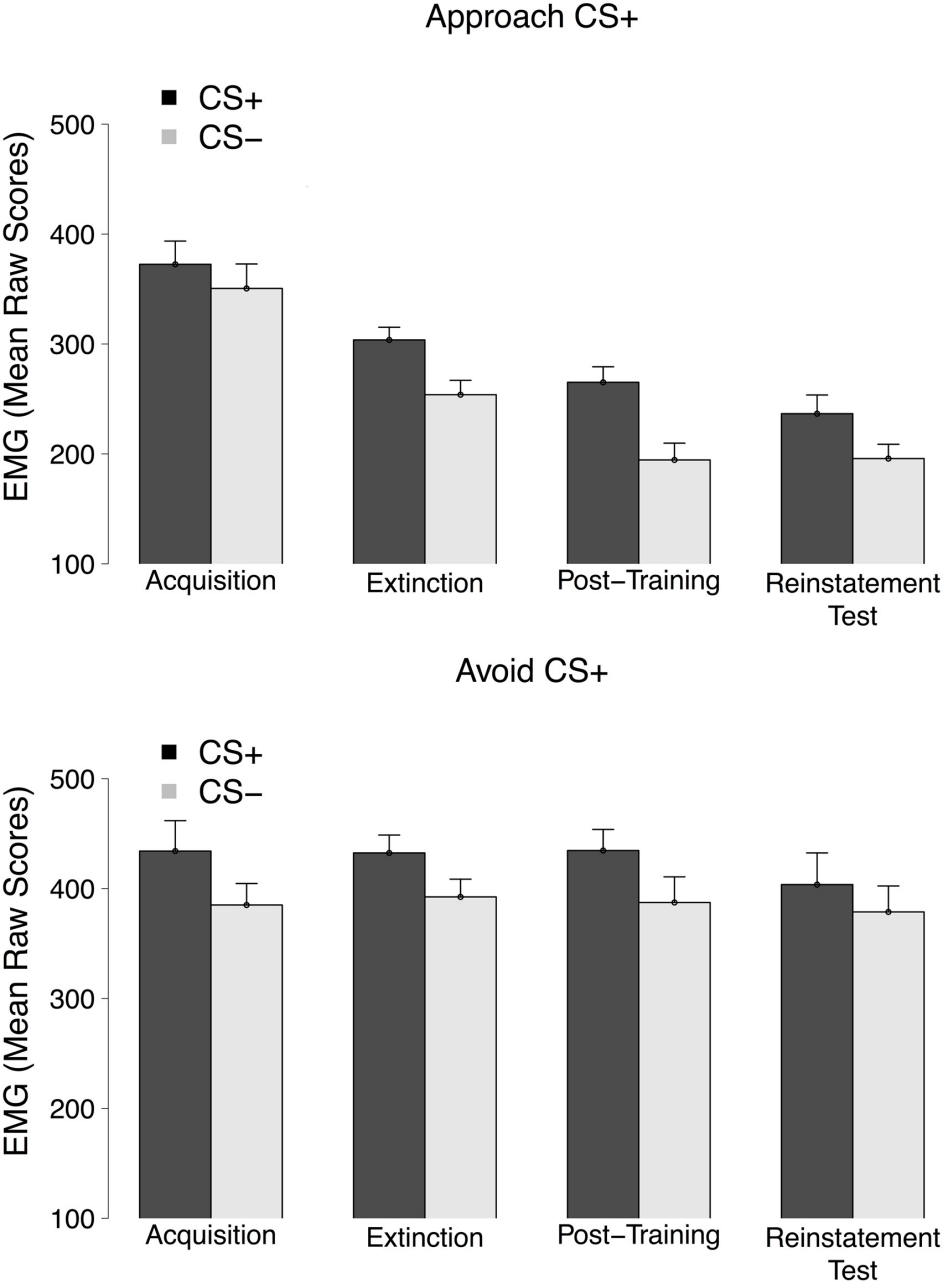
Mean FPS to the CS^+^ and the CS^−^ trials across all conditioning phases for the Approach CS^+^ (top panel) and Avoid CS^+^ (bottom panel) group. NA trials are not depicted. Error bars were computed using the approach described in [21].

Fig 4 depicts FPS responses to each CS throughout the conditioning phases. During the acquisition phase, startle potentiation was higher for the CS^+^ compared to the CS^−^, main effect of Stimulus, *F*(1, 38) = 9.21, *p* <.01, $\eta_{p}^{2}=.21$, and this similarly for both groups, Stimulus × Group interaction, *F*(1, 38) = 1.31, *p* = .26, $\eta_{p}^{2}=.03$, indicating the successful acquisition of differential FPS.

FPS differentiation remained in the extinction phase, main effect of Stimulus, *F*(1, 38) = 15.50, *p* <.001, $\eta_{p}^{2}=.29$, again similar for both groups, Stimulus × Group interaction, *F* < 1. No decrease in differential startle responses was observed between the acquisition and the extinction phase, Stimulus × Phase interaction, *F* < 1, for either group, Stimulus × Phase × Group interaction, *F*(1, 38) = 2.00, *p* = .17, $\eta_{p}^{2}=.05$.

Differential FPS responses did not change from the extinction to the post-training test phase either, Stimulus × Phase interaction, *F* < 1, Stimulus × Phase × Group interaction, *F* < 1. Differential FPS responding was thus preserved during the post-training phase, main effect of Stimulus, *F*(1, 38) = 10.37, *p* <.01, $\eta_{p}^{2}=.20$, and was comparable for both groups, Stimulus × Group interaction, *F* < 1.

Differential FPS responses did not seem to change from the extinction to the reinstatement test phase either, Stimulus × Phase interaction, *F* < 1, Stimulus × Phase × Group interaction, *F* < 1. Within the reinstatement test phase, differential FPS potentiation was observed, main effect of Stimulus, *F*(1, 38) = 4.40, *p* = .04, $\eta_{p}^{2}=.10$, in the absence of a between-groups difference, Stimulus × Group interaction, *F* < 1.

### Skin conductance response data

**Fig 5:**
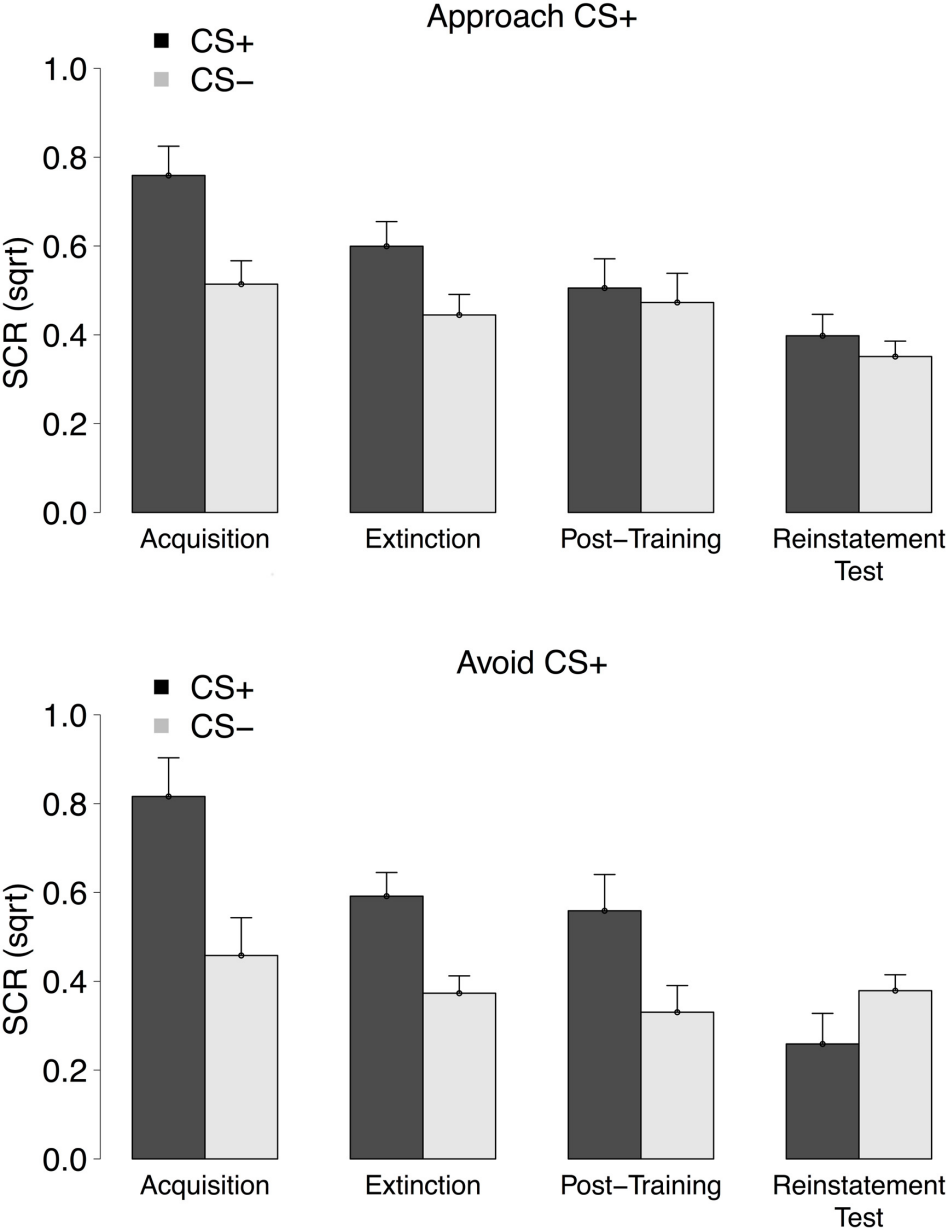
Mean SCRs to the CS^+^ and CS^−^ trials during all conditioning phases for the Approach CS^+^ (top panel) and the Avoid CS^+^ (bottom panel) group. Error bars were computed using the approach described in [21].

Fig 5 depicts mean SCRs for the CS^+^ and the CS^−^. During the acquisition phase, participants’ SCRs were higher for the CS^+^ compared to the CS^−^ trials, main effect of Stimulus, *F*(1, 38) = 42.92, *p* <.001, $\eta_{p}^{2}=.53$, a result that was similar across groups, Stimulus × Group interaction, *F*(1, 38) = 1.52, *p* = .23, $\eta_{p}^{2}=.04$. Those results suggest the successful acquisition of differential SCRs for both groups.

SCR differentiation was also observed in the extinction phase, main effect of Stimulus, *F*(1, 38) = 26.31, *p* <.001, $\eta_{p}^{2}=.41$, for both groups, Stimulus × Group interaction, *F* < 1. However, this differentiation tended to be smaller than in the acquisition phase, Stimulus × Phase interaction, *F*(1, 38) = 3.78, *p* = .06, $\eta_{p}^{2}=.09$, for both groups, Stimulus × Phase × Group interaction, *F* < 1, indicating the successful reduction of differential SCRs.

SCRs towards the CSs were similar between the extinction and the post-training test phase, Stimulus × Phase interaction, *F* < 1, Stimulus × Phase × Group interaction, *F* < 1. SCR differentiation was still present in the post-training test phase, *F*(1, 38) = 3.96, *p* = .05, $\eta_{p}^{2}=.09$, for both groups, Stimulus × Group interaction, *F*(1, 38) = 2.23, *p* = .14, $\eta_{p}^{2}=.06$.

The change in SCR differentiation between the extinction and the reinstatement test phase was different between groups, Stimulus × Phase × Group interaction, *F*(1, 38) = 3.95, *p* = .05, $\eta_{p}^{2}=.09$. Separate within-group analyses demonstrated that there was a change in differential SCRs from the extinction to the reinstatement test phase for the Avoid CS^+^ group, Stimulus × Phase interaction, *F*(1, 17) = 11.09, *p* <.01, $\eta_{p}^{2}=.40$, but not for the Approach CS^+^ group, Stimulus × Phase interaction, *F*(1, 21) = 2.84, *p* = .11, $\eta_{p}^{2}=.12$. Quite unexpectedly, in the Avoid CS^+^ group, there was a significant reduction in SCR from the extinction to the reinstatement test phase, *F*(1, 17) = 10.45, *p* = .01, $\eta_{p}^{2}=.38$, for the CS^+^, and no significant differences in SCR for the CS^−^, *F* < 1. Lastly, no differential SCRs were observed within the reinstatement test phase, main effect of Stimulus, *F* < 1, for either group, Stimulus × Group interaction, *F*(1, 38) = 3.07, *p* = .09, $\eta_{p}^{2}=.08$.

### Manikin AAT data

**Fig 6:**
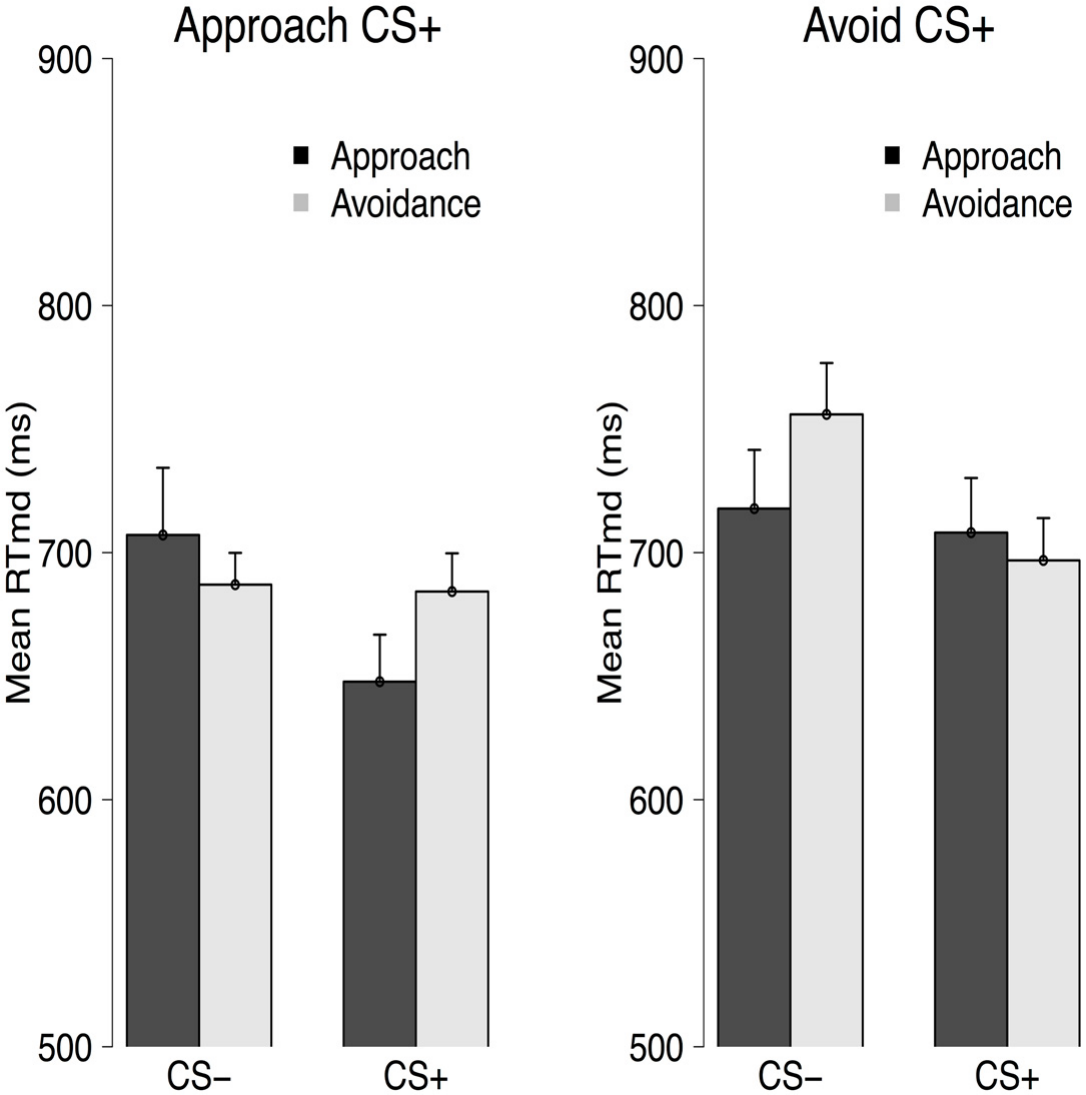
Mean median RT in ms for the Approach CS^+^ (left panel) and the avoid CS^+^ (right panel) groups for the manikin AAT. Error bars were computed using the approach described in [21].

No between-group differences were found for approaching and avoiding the different stimuli, Stimulus × Response × Group interaction, *F*(1, 38) = 2.43, *p* = .13, $\eta_{p}^{2}=.06$ (see Fig 6). Careful inspection of the data using combined scores—the combined scores were computed as the difference between the medians in the incongruent trials (approach CS^−^ and avoid CS^+^) from the congruent trials (approach CS^+^ and avoid CS^−^)—showed that this was due to two participants scoring 2.5 standard deviations above the mean. After repeating the analyses without those outlying values, the between-group interaction approached significance, *F*(1, 36) = 3.58, *p* = .07, $\eta_{p}^{2}=.09$, with participants in the Avoid CS^+^ group tending to be faster to avoid the CS^+^ and approach the CS^−^ than the reverse, Stimulus × Response interaction, *F*(1, 16) = 4.00, *p* = .06, $\eta_{p}^{2}=.20$, whereas a non-significant pattern in the opposite direction emerged for the Approach CS^+^ group, Stimulus × Response interaction, *F* < 1. Those results suggest that there was transfer from the training AAT (joystick task) to the assessment AAT. For the interested reader, we report the results of all the previous analyses after excluding the data of these two participants in S1 Text. In summary, the results from the additional analyses replicate the main results with two exceptions: 1) there was a non-significant trend for a Stimulus × Response × Group interaction for the Manikin AAT (see main text) and 2) in contrast to the main analyses, the Stimulus × Phase × Group interaction for SCR failed to reach significance.

### CS-evaluation data

Participants evaluated the CS^+^ as more unpleasant than the CS^−^, *F*(1, 38) = 71.76, *p* <.001, $\eta_{p}^{2}=.65$. That effect was similar across groups, Stimulus × Group interaction, *F*(1, 38) = 2.65, *p* = .11, $\eta_{p}^{2}=.07$ (see Table 1).

## Discussion

We investigated whether supplementing a Pavlovian extinction procedure with an approach training procedure would result in enhanced fear reduction and/or would prevent the return of conditioned fear. Although the approach training was effective in changing approach latencies within the joystick task, and despite some evidence for generalization of the training effects to approach-avoidance latencies in a manikin AAT, no between-group differences in differential fear responding during either the post-training or the reinstatement phase were found, be it in a subjective measure (i.e., US expectancy ratings) or in the physiological indices of fear (i.e., FPS and SCR) that we recorded. In summary, our results suggest weak if any *near transfer* of the approach-avoidance training to a manikin AAT task but no *far transfer* to subjective and physiological indices of conditioned fear.

We used the manikin AAT for assessing approach-avoidance tendencies towards the CSs. Results showed that the approach training led to (weak) training-compatible effects on approach-avoidance tendencies, with participants in the Approach CS^+^ group being generally faster to approach the CS^+^ and avoid the CS^−^ than the reversed, and participants in the Avoid CS^+^ group showing the opposite pattern. One might claim that those changes in approach-avoidance tendencies are by themselves indicative of a partially successful change in fear levels. Indeed, action tendencies are an integral part of emotions in general, and avoidance tendencies are a key component of fear [22, 23]. Accordingly, it could be argued that fear levels are diminished if avoidant action tendencies are reduced, even if other dimensions of fear (e.g., threat expectancy or physiological responding) remain unaffected. This is not merely a theoretical argument. Clinically, one of the defining features of pathological fear and anxiety disorders is excessive avoidance towards seemingly innocuous cues or situations [1]. The modification of action tendencies, even if not accompanied by a reduction in other fear symptoms, could potentially prove beneficial in clinical practice. However, it is an open question whether modified action tendencies after a training AAT would also translate in sustainable changes in overt avoidance behaviour, especially if not accompanied by changes in other fear systems. According to dual-process models of behaviour (e.g., [24]), overt behaviour results from a complex interplay between impulsive action tendencies (generated by an implicit system) and cognitive control processes (exerted by a reflective system), so there does not need to be a one-to-one relation between reduced avoidant action tendencies and reduced behavioural avoidance. The extent to which changes in impulsive action tendencies towards fear cues, e.g., after exposure, uniquely predict changes in overt avoidance is a topic of ongoing research in our lab and elsewhere.

Of note, no complete extinction was observed for any of the physiological or subjective measures. There are at least two candidate explanations for this result. First, we analysed physiological and subjective rating data using the mean responses across each experimental phase; full extinction would be expected towards the end of the extinction phase only, if at all. Second, we intentionally limited the number of extinction trials. In previous work, we used 20 extinction trials rather than 12 (e.g., [19]). Here, we restricted the number of extinction trials to leave room for a further reduction of fear responding through the training AAT and to minimize habituation to the startle probe.

Relative to the moderate levels of differential fear responding still observed after extinction training, significant return of fear was not observed even in the Avoid CS^+^ group. Failure to obtain differential reinstatement after extinction training is far from uncommon [25]. While the lack of reinstatement obtained here may have left limited room for approach training to attenuate return-of-fear (it is hard to attenuate something that is absent), the considerable differential responding preserved during extinction did provide ample room for approach training to enhance extinction effects; no such enhancement was observed at all.

We refrained from collecting CS evaluation ratings after the joystick AAT to prevent a potential influence of such rating phase on our primary measures of interest (i.e., EMG, SCR, US-expectancies). The absence of those ratings implies that we have no direct evidence to ascertain that the joystick AAT modified the CS evaluations as intended.

In terms of intervention, then, our findings provide little encouragement for the addition of approach-avoidance bias modification training to extinction-based therapies for anxiety disorders. Approach training may be a tool for the modification of dysfunctional avoidance tendencies towards phobic cues but appears to have limited if any impact on other fear responses; whether the modification of approach-avoidance tendencies would have any influence on overt avoidance awaits further research.

## Supplementary Material

## Supplementary Analyses after Deleting Participants Scoring 2.5 Standard Deviations Above the Mean in the Manikin AAT

### Self-report data

Groups did not differ in terms of sex (Pearson *χ* ^2^ = .84, *p* = .39), state or trait anxiety, anxiety sensitivity, selected US level or US evaluations (all *ts* < 1.4).

### Training AAT data

Results from the joystick AAT revealed that the between-groups difference in response pattern was different between the baseline and the test phase, Stimulus × Response × Phase × Group interaction, *F*(1, 36) = 8.58, *p* < 0.01, $\eta_{p}^{2}=.19$. Unexpectedly, a non-significant trend for between-groups differences was present at baseline, *F*(1, 36) = 3.26, *p* = .08, $\eta_{p}^{2}=.08$, reflecting a baseline difference that, if anything, should work against our hypothesis. A significant between-groups difference in the opposite direction was obtained in the test phase, Stimulus × Response × Group interaction, *F*(1, 36) = 4.37, *p* = .04, $\eta_{p}^{2}=.11$, with participants in the Approach CS^+^ group now being relative faster in approaching the CS^+^ and avoiding the CS^−^, and participants in the Avoid CS^+^ group in avoiding the CS^+^ and approaching the CS^−^.

Taken together, our results indicate that the approach training was successful.

### US-expectancy rating data

During the acquisition phase, participants in both groups learned to expect the US after the CS^+^ and not after the CS^−^, main effect of Stimulus, *F*(1, 36) = 469.90, *p* <.001, $\eta_{p}^{2}=.93$, Stimulus × Group interaction, *F* < 1, suggesting similarly successful acquisition of differential US-expectancy in both groups.

Differential US-expectancy was partially preserved in the extinction phase, main effect of Stimulus, *F*(1, 36) = 78.97, *p* <.001, $\eta_{p}^{2}=.69$, and this to a similar extent in both groups, Stimulus × Group interaction, *F* < 1. Nonetheless, compared to the acquisition phase, there was a significant decline in differential US-expectancy, Stimulus × Phase interaction, *F*(1, 36) = 48.75, *p* <.001, $\eta_{p}^{2}=.58$, across Groups, *F* < 1.

Differential US-expectancy seemed to further decline from the extinction to the post-training test phase as suggested from a non-significant trend for a Stimulus × Phase interaction, *F*(1, 36) = 3.73, *p* = .06, $\eta_{p}^{2}=.10$, which was comparable between groups, Stimulus × Phase × Group interaction, *F* < 1. Specifically, there a significant reduction in US-expectancy for the CS^+^, CS^+^ main effect of Phase, *F*(1, 36) = 4.11, *p* = .05, $\eta_{p}^{2}=.10$, and no change for the CS^−^, CS^−^ main effect of Phase, *F* < 1. Differential US-expectancy remained present throughout the post-training test phase, main effect of Stimulus, *F*(1, 36) = 46.55, *p* <.001, $\eta_{p}^{2}=.56$, for both groups, Stimulus × Group, *F* < 1.

Differential US-expectancy again declined from the extinction to the reinstatement test phase, Stimulus × Phase interaction, *F*(1, 36) = 23.53, *p* <.001, $\eta_{p}^{2}=.40$, in a similar fashion for both groups, Stimulus × Phase × Group interaction, *F* < 1.12. This change again stemmed from a reduction in US-expectancy for the CS^+^, Stimulus × Phase interaction, *F*(1, 36) = 19.29, *p* <.001, $\eta_{p}^{2}=.35$, with no change for the CS^−^, Stimulus × Phase interaction, *F* < 1. Lastly, differential US-expectancy remained present in the reinstatement test phase, main effect of Stimulus, *F*(1, 36) = 20.32, *p* <.001, $\eta_{p}^{2}=.36$, for both groups, Stimulus × Group interaction, *F* < 1.

### FPS data

During the acquisition phase, startle potentiation was higher for the CS^+^ compared to the CS^−^, main effect of Stimulus, *F*(1, 36) = 8.26, *p* <.01, $\eta_{p}^{2}=.19$, and this similarly for both groups, Stimulus × Group interaction, *F* < 1.12, indicating the successful acquisition of differential FPS.

FPS differentiation remained in the extinction phase, main effect of Stimulus, *F*(1, 36) = 15.17, *p* <.001, $\eta_{p}^{2}=.30$, again similar for both groups, Stimulus × Group interaction, *F* < 1. No decrease in differential startle responses was observed between the acquisition and the extinction phase, Stimulus × Phase interaction, *F* < 1, for either group, Stimulus × Phase × Group interaction, *F*(1, 36) = 2.15, *p* = .15, $\eta_{p}^{2}=.06$.

Differential FPS responses did not change from the extinction to the post-training test phase either, Stimulus × Phase interaction, *F*(1, 36) = 1.43, *p* = .24, $\eta_{p}^{2}=.04$, Stimulus × Phase × Group interaction, *F* < 1. Differential FPS responding was thus preserved during the post-training test phase, main effect of Stimulus, *F*(1, 36) = 13.34, *p* <.001, $\eta_{p}^{2}=.27$, and was comparable for both groups, Stimulus × Group interaction, *F* < 1.

Differential FPS responses did not seem to change from the extinction to the reinstatement test phase either, Stimulus × Phase interaction, *F*(1, 36) = 1.14, *p* = .29, $\eta_{p}^{2}=.03$, Stimulus × Phase × Group interaction, *F* < 1. Within the reinstatement test phase, differential FPS potentiation was almost observed, main effect of Stimulus, *F*(1, 36) = 3.26, *p* = .08, $\eta_{p}^{2}=.08$, in the absence of a between-groups difference, Stimulus × Group interaction, *F* < 1.2.

### Skin conductance response data

During the acquisition phase, participants’ SCRs were higher for the CS^+^ compared to the CS^−^ trials, main effect of Stimulus, *F*(1, 36) = 40.40, *p* <.001, $\eta_{p}^{2}=.53$, a result that was similar across groups, Stimulus × Group interaction, *F*(1, 36) = 1.40, *p* = .24, $\eta_{p}^{2}=.04$. Those results suggest the successful acquisition of differential SCRs for both groups.

SCR differentiation was still present in the extinction phase, main effect of Stimulus, *F*(1, 36) = 24.60, *p* <.001, $\eta_{p}^{2}=.41$, for both groups, Stimulus × Group interaction, *F*(1, 36) = 1.58, *p* = .22, $\eta_{p}^{2}=.04$. However, this differentiation was smaller than in the acquisition phase, Stimulus × Phase interaction, *F*(1, 36) = 4.42, *p* = .04, $\eta_{p}^{2}=.11$, for both groups, Stimulus × Phase × Group interaction, *F* < 1, indicating the successful reduction of differential SCRs.

SCRs towards the CSs were similar between the extinction and the post-training test phase, Stimulus × Phase interaction, *F* < 1, Stimulus × Phase × Group interaction, *F* < 1. SCR differentiation was still present in the post-training test phase as suggested by a non-significant trend for a main effect of stimulus, *F*(1, 36) = 3.54, *p* = .07, $\eta_{p}^{2}=.09$, which was similar across groups, Stimulus × Group interaction, *F*(1, 36) = 1.32, *p* = .26, $\eta_{p}^{2}=.04$.

There was a change in SCR differentiation between the extinction and the reinstatement test phase, Stimulus × Phase interaction, *F*(1, 36) = 13.44, *p* <.001, $\eta_{p}^{2}=.27$, which was similar across groups, *F*(1, 36) = 2.32, *p* = .14, $\eta_{p}^{2}=.06$. Specifically, and in contrast to the extinction phase, no SCR differentiation was present in the reinstatement test phase, main effect of stimulus, *F* < 1, across groups, Stimulus × Group interaction, *F* < 1.1. This differentiation was due to a significant decrease in SCR responding towards the CS^+^, CS^+^ main effect of Phase, *F*(1, 36) = 16.68, *p* <.001, $\eta_{p}^{2}=.32$, and stable SCR responding towards the CS^−^ across phases, CS^−^ main effect of Phase, *F*(1, 36) = 1.68, *p* = .20, $\eta_{p}^{2}=.05$.

### Manikin AAT data

Please see the main article.

### CS-evaluation data

Participants evaluated the CS^+^ as more unpleasant than the CS^−^, *F*(1, 36) = 69.66, *p* <.001, $\eta_{p}^{2}=.66$. That effect was similar across groups, Stimulus × Group interaction, *F*(1, 36) = 1.42, *p* = .24, $\eta_{p}^{2}=.04$.
